# crisprSQL: a novel database platform for CRISPR/Cas off-target cleavage assays

**DOI:** 10.1093/nar/gkaa885

**Published:** 2020-10-21

**Authors:** Florian Störtz, Peter Minary

**Affiliations:** Department of Computer Science, University of Oxford, Parks Road, Oxford OX1 3QD, UK; Department of Computer Science, University of Oxford, Parks Road, Oxford OX1 3QD, UK

## Abstract

With ongoing development of the CRISPR/Cas programmable nuclease system, applications in the area of *in vivo* therapeutic gene editing are increasingly within reach. However, non-negligible off-target effects remain a major concern for clinical applications. Even though a multitude of off-target cleavage datasets have been published, a comprehensive, transparent overview tool has not yet been established. Here, we present crisprSQL (http://www.crisprsql.com), an interactive and bioinformatically enhanced collection of CRISPR/Cas9 off-target cleavage studies aimed at enriching the fields of cleavage profiling, gene editing safety analysis and transcriptomics. The current version of crisprSQL contains cleavage data from 144 guide RNAs on 25,632 guide-target pairs from human and rodent cell lines, with interaction-specific references to epigenetic markers and gene names. The first curated database of this standard, it promises to enhance safety quantification research, inform experiment design and fuel development of computational off-target prediction algorithms.

## Graphical Abstract

**Graphical Abstract:**
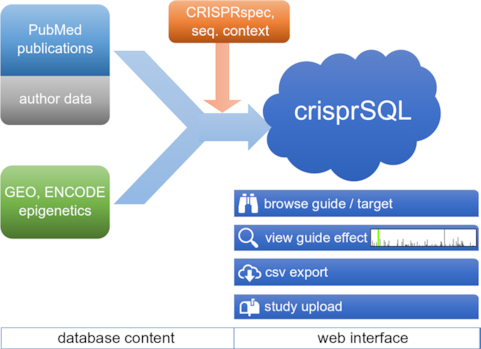
Overview of the crisprSQL database. Cleavage and epigenetics data (left) are joined together and supplemented by attributes calculated from their properties (top). This forms the crisprSQL database, which has an online user interface supporting data browsing, export and upload of new studies (right).

## INTRODUCTION

Several clinical applications of the CRISPR/Cas gene editing technology have been realised to date, such as personalised cancer treatment with genetically modified chimeric antigen receptor (CAR) T cell-based therapies (1). A variety of possible therapeutic applications in the fields of genetic diseases, infectious diseases and cancers are being investigated (2), as exemplified by 38 clinical studies referring to CRISPR currently registered on http://clinicaltrials.gov (accessed 7 August 2020). As Dai *et al.* state, ‘one of the major hurdles to the clinical translation of CRISPR/Cas9 is its off-target effects’ (3), referring to the unwanted nucleotide insertions or deletions created by CRISPR outside of the targeted genomic locus. Electing an appropriate guide RNA (gRNA) for the desired DNA target sequence is considered a ‘crucial first step in avoiding off-target effects’ (4). In order to mitigate off-target effects, ‘a set of validated off-target sites should be compiled and used to create a reagent set to quantitatively study off-target effects via NGS methods’ (5). With off-target data spread across supplementary data of various publications, this remains a tedious and time-consuming task.

Besides the design and execution of medical studies, a comprehensive database of well-known off-target interactions is required for the validation of novel off-target detection methods (6) and new implementations of existing methods in a laboratory setting.

The development of computational off-target prediction algorithms (7,8) also relies on the presence of a comprehensive dataset quantifying off-target effects of gRNAs on DNA from certain cell lines, which is usually annotated with a selection of epigenetic markers by the respective authors. The varying choice of annotation sources, training data and test procedures can result in issues of reproducibility (9).

We contribute an online database which promises to enhance clinical, laboratory and computational research by providing the first one-stop source of off-target resolved CRISPR/Cas cleavage data to date, accompanied by epigenetic annotations and visualisations. In order to provide maximum value for a range of fields including transcriptomics, gene knockout experiments, editing safety-driven guide design and cleavage efficiency prediction, crisprSQL offers sequence-resolved data whilst also bridging the gap to gene resolution by attaching GENCODE gene names to interaction targets.

## MATERIALS AND METHODS

### Data acquisition

#### Cleavage rate sources & formats

There have been numerous publications scrutinising the off-target effects of *Streptococcus pyogenes* CRISPR/Cas9 over the past decade (see Table 1), most of which rely on second-generation whole-genome sequencing tools. First-generation approaches such as the T7 endonuclease assay only find off-targets among a previously *in silico* defined set, therefore only yielding limited insight into the full off-target profile.

In order to provide the most detailed insight into cleavage processes, we have chosen to include only those studies which offer base pair-resolved on- and off-target, as well as an unambiguous assignment of which specific guide caused a given cleavage event. This excludes pure on-target studies, pure gene knock-out studies and most studies done on whole gRNA libraries. To our best knowledge, this is an objective which has not been realised before. The use of ENCODE Tier 1 immortalised cell lines (10) facilitates comparison between experiments and annotation with cell-specific metadata.

We manually extracted the cleavage frequencies from the references in Table 1 using an online PDF-to-csv tool wherever these were not supplied in text form. The included publication (23) chose identical target sequences in both open and closed chromatin regions, thereby promising very telling data on the effect of chromatin accessibility. Publication (28) which is included in the CRISPOR dataset (27) was excluded here because extraction of the relevant data (gRNA, target locus or sequence, binding & cleavage frequency) was not unambiguously possible. For some studies, we additionally obtained unpublished data points from the same measurement series which authors were happy to share on request.

**Table 1.** Data sources included in crisprSQL (version 10 July 2020), chronologically ordered by publication date

Ref.	Technique	Detects	Guides	Targets	Annot.	Assembly	Cell lines
(11)	T7E1	Heterodupl. DNA	9	88	0	hg19	U2OS, HEK293, K562
(12)	Targeted PCR	Genome change	10	116	11	hg19	K562
(6)	Guide-seq	DSBs	12	575	381	hg19	U2OS, HEK293
(13)	Digenome-seq	Genome change	2	162	7	hg19	HAP1, K562
(14)	BLESS, ChIP	DSBs	6	87	53	hg19, mm9	293FT, N2a
(15)	HTGTS	DNA junctions	3	87	87	hg19	HEK293T
(16)	IDLVs	Viral integration	1	13	13	hg19	HEK293T
(17)	Digenome-seq	Genome change	10	258	234	hg19	HeLa
(18)	Guide-seq	DSBs	7	61	28	hg19	U2OS
(19)	BLESS	DSBs	3	31	31		HEK
(20)	CIRCLE-seq	DSBs	18	7374	3493	hg19	HEK293, U2OS, K562
(21)	Guide-seq	DSBs	5	203	107	hg19	U2OS
(22)	SITE-Seq	DSBs	8	1630	210	hg38	HEK293
(23)	DIG-seq	Genome change	7	141	132	hg19	HeLa
(24)	Guide-seq, WGS	DSBs	31	426	0	mm9, rn5	Mouse & rat embryos
(25)	Guide-seq	DSBs	10	272	160	hg19	U2OS
(26)	NucleaSeq	Cleaved products	2	14108	0		Custom DNA library
		Sum	144	25632	4947		

With 25,632 guide-target pairs, our dataset is more than an order of magnitude larger than both the CRISPOR (27) and DeepCRISPR (7) off-target datasets with less than 680 guide-target pairs each. Note that we have excluded guides with less than two reported off-targets, but kept GC-rich guides and guide-target pairs with low cleavage frequencies. The ‘annotated’ column counts how many of the respective guide-target pairs are annotated with at least one epigenetic marker. The term ‘target’ includes both on-targets, i.e. the intended cutting site which is homologous to the guide sequence, and mismatched off-targets.

The off-target cleavage assays included in crisprSQL can be divided into two categories. DSB capture approaches in a cellular context (*in situ*) are more physiologically relevant, albeit prone to false positives due to cellular processes which may generate non-Cas9 related DSBs. IDLV (16) and GUIDE-seq (6) rely on the non-homologous end joining-mediated integration of sequence markers into sites upon DSB. IDLV integration does not always happen at the exact DSB location, whilst GUIDE-seq is limited to blunt-end DSBs (29). Typical sensitivities range from 0.1% (6,15) to 1% (16). Other *in situ* techniques include HTGTS (15) (translocation of DSBs to bait DSBs) and BLESS (14) (integration of biotinylated linkers).

*In vitro* approaches outside of a cellular context do not include effects of epigenetic factors and chromatin on DSB formation, making the subsequent validation of observed off-targets in cells desirable. SITE-Seq (22) has been shown to overestimate off-targets relative to those observed in a cellular context. CIRCLE-seq (20) shows high sensitivity through enrichment of Cas9-cleaved genomic DNA, which leads to a considerable level of background noise overshadowing true positive off-targets (29). DIG-seq (23) uses chromatin-associated DNA and can therefore assess the influence of chromatin on Cas9 cleavage. Experimental protocols for the different assays entail different relative concentrations of Cas9 expression plasmid (100 ng–1 μg), gRNA-encoding plasmid (50 ng–1 μg), and possible additions such as blasticidin resistance-inducing plasmids. The ratio of Cas9 plasmid : gRNA plasmid ranges from 1:1 to 3:1, and the time between Cas9 transfection and cell harvest for DSB detection ranges from 24 h (14) to 5 days (24).

With the exception of the NucleaSeq assay (26), crisprSQL only includes data points which have been validated in cells. For *in vitro* methods, this is a certain fraction of DSB sites identified by the respective assay, ranging from 62% (24) to 100% (13,22). For these methods, validation data in cellular context has been gained through targeted deep sequencing.

It has become convention to give guide and target sequences as their respective complement in the protospacer strand such that a canonically matched guide-target pair shows as identical base letters. To extract the actual binding sequences from our database, one therefore has to exchange thymine for uracil in the saved gRNA sequence, and take the canonical complement of the saved target sequence.

#### Data processing

The resulting csv file was imported in Python using pandas. Missing genomic locus information was filled in using the blastn tool. For sequences which were shorter than 23 bp, samtools faidx was used to extend them to this length, making sure the found sequence contains at least the 10 PAM-proximal base pairs of the original, has no uncalled bases (N) and an identical PAM. It has been reported that sequence context plays a significant role for cleavage efficiency (27,30). We therefore provide the 169 bp sequence context around the centre of each target obtained using samtools faidx. This length has been chosen such that the nucleosome core DNA length of 147 bp can be retrieved around any of the 23 bp of each target. To annotate targets with the matching gene name, we retrieve the gff3 files for the respective genomes from the GENCODE database (31), extract the gene annotations and convert them to bed format. Overlap is then checked for each target sequence.

#### Delivery method

In order to be able to scrutinise the effect of the delivery method of the CRISPR machinery into cells, the database contains a column characterising the mode of delivery (lipofection/electroporation/other), and a column indicating whether a given data point was gained in a cellular context via an unbiased (genome-wide) detection method.

#### Epigenetic factors

Binding and cleavage as natural events do not only depend on sequence information as acquired in the first step, but also on further atomistic parameters of the guide–target heteroduplex. These epigenetic factors have been obtained experimentally using a variety of techniques. The ENCODE database (32) offers a platform which holds this experimental data and allows to search it. SCREEN is a search tool for the ENCODE database which summarises the presence of certain epigenetic factors / candidate regulatory elements along the human (hg19) and mouse (mm10) genome, for several cell lines or tissues. We obtained bed files from the SCREEN web interface which contain genomic regions in which epigenetic markers are present for four individual markers. This choice has become customary in the field of computational off-target prediction (7,33,34): (a) CTCF: CCCTC-binding factor, present at sites which activate gene transcription and are related to chromatin organisation (35), (b) DNase-seq: sites sensitive to the DNase I enzyme correspond to open chromatin (i.e. accessibility of the DNA strand), (c) RRBS: DNA methylation state, obtained by converting unmethylated cytosines to uracil, (d) H3K4me3: trimethylation of the lysine-4 on the histone H3 protein, associated with transcription of nearby genes (36). A full list of the assay files which we used can be found in Supplementary Table S2. We only considered the regions where the marker value is in the 95th percentile across the genome by choosing a cutoff of *Z* ≥ 1.64 on the SCREEN web page. Genomic regions present in our database which overlap with a marked region in one of these bed files are annotated with the respective *Z* score, as well as the respective SCREEN accession string to ensure traceability. Overlap is assessed using the bedtools intersect command.

Since SCREEN only offers this data for the hg19 and mm10 gene assemblies and a selection of cell lines, data for other reference genomes was obtained from the ENCODE database itself. The fifth epigenetic marker came from the DRIP-seq assay (37), which detects R-loops between DNA and RNA formed during transcription, exposing single-stranded DNA and thereby possibly causing genome instability. Data files for the DRIP assay had to be converted from bigWig to bed using the BEDOPS suite (38). We downloaded further data gained through the DRIP assay in bed format from the publications (37,39–41) via the GEO database (42). Target loci for which an epigenetic overlap has been found using BamTools (43) were updated using the quality score of the respective bam file region(s) and the unique ENCODE accession string of the bam file. Where two or more overlaps were found for a given target region, only the maximum quality score was kept.

We should note that not all epigenetic markers are available for every cell type and genome assembly (see Table 2). As a workaround, sequences could be realigned to other genome assemblies within species (i.e. hg38 to hg19, mm9 to mm10). As this might introduce impurity into the data, it has not been implemented. However, with the data from Table 2, we are able to check annotation for more than 97% of the off-target loci with at least two of five epigenetic markers.

**Table 2.** Availability of epigenetic markers by cell line in the ENCODE, SCREEN (32) and GEO (42) databases

		Epigenetic marker				
Assembly	Cell line	DNase	CTCF	H3K4me3	RRBS	DRIP
hg19	HEK293	$\checkmark$	$\checkmark$	$\checkmark$	$\checkmark$	$\checkmark$
	K562	$\checkmark$	$\checkmark$	$\checkmark$	$\checkmark$	$\checkmark$
	HeLa	$\checkmark$	$\checkmark$	$\checkmark$	$\checkmark$	$\checkmark$
	U2OS	$\checkmark$	$\checkmark$	$\checkmark$		$\checkmark$
	HAP1	$\checkmark$	$\checkmark$	$\checkmark$		
hg38	HEK293	$\checkmark$	$\checkmark$	$\checkmark$		
	K562	$\checkmark$	$\checkmark$	$\checkmark$		
	HeLa	$\checkmark$	$\checkmark$	$\checkmark$		
	U2OS					
	HAP1	$\checkmark$				
mm10	Embryonic tissue	$\checkmark$		$\checkmark$		
mm9	N2a					
rn5	Embryonic tissue					

#### Interaction energies

In reference (44), the authors present an approximate binding energy model (termed CRISPRspec) for the Cas9–gRNA–DNA complex, including contributions from the gRNA–DNA hybridisation and the opening of the DNA–DNA duplex in the target region. RNAfold (45) is used to calculate the approximate free energy of the gRNA folding based on sequence information. We calculate a representative choice of five energy contributions for each gRNA–target sequence pair and annotate the respective data point. The function arguments used to retrieve these can be found in Supplementary Table S1.

### Data storage

**Figure 1.**
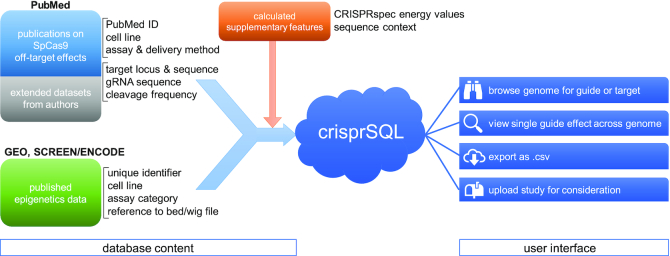
Overview of the crisprSQL database. Cleavage and epigenetics data (left blue/green fields) are joined together and supplemented by attributes calculated from their properties (top orange field). Additional cleavage data obtained from the authors was included where possible. Brackets indicate single data tables. This forms the crisprSQL database, which has an online user interface supporting data browsing, export and upload of new studies (right).

In order to achieve high enough read and write speeds, we use a Python-implemented SQLite database in local memory for data storage and augmentation. The database is internally separated into three data tables which contain information about the included cleavage assay publications, epigenetics studies datasets and measured guide-target cleavage data points, respectively. This ensures that we are able to save all necessary information in a structured and efficient way which allows full traceability (see Figure 1). From this dataset, we are able to extract convenient views, e.g. in csv format for import into third party software, or in MySQL format to provide a website interface.

In conjunction with external tools, our database can be used to suggest optimal guides for cleavage at a certain locus whilst minimising off-target effects.

### Website interface

#### Implementation

In order to make our database easily accessible, we implemented a web-interface using a MySQL-based instance of our database. The frontend and search function are rendered using PHP.

An appropriate subset of database columns was chosen for this so as to not overload the user interface. Columns describing the CRISPRspec energy contributions and the sequence context are not included in the web interface, only in the csv file. Furthermore, the website shows only the presence or absence of a nonzero epigenetic score but not its value. The web interface offers a full-text search for specific guides, target regions or cell lines, and a download of the full database in csv format. Similar services already exist for high-throughput on-target studies (46), but to our best knowledge, crisprSQL is the first online database for Cas9 off-target assays.

**Figure 2.**
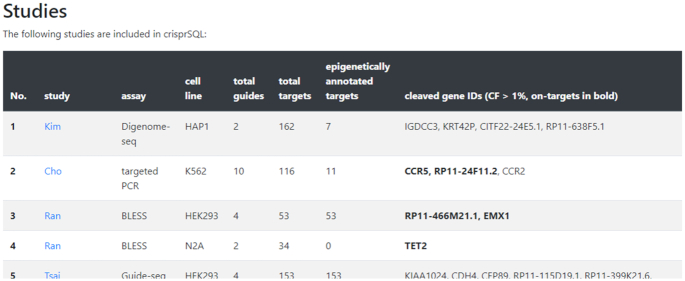
Overview of the included studies, together with metadata such as assay type, the total number of guides and guide-target pairs observed as well as the predominantly cleaved gene names. Studies have been split up according to cell lines.

**Figure 3.**
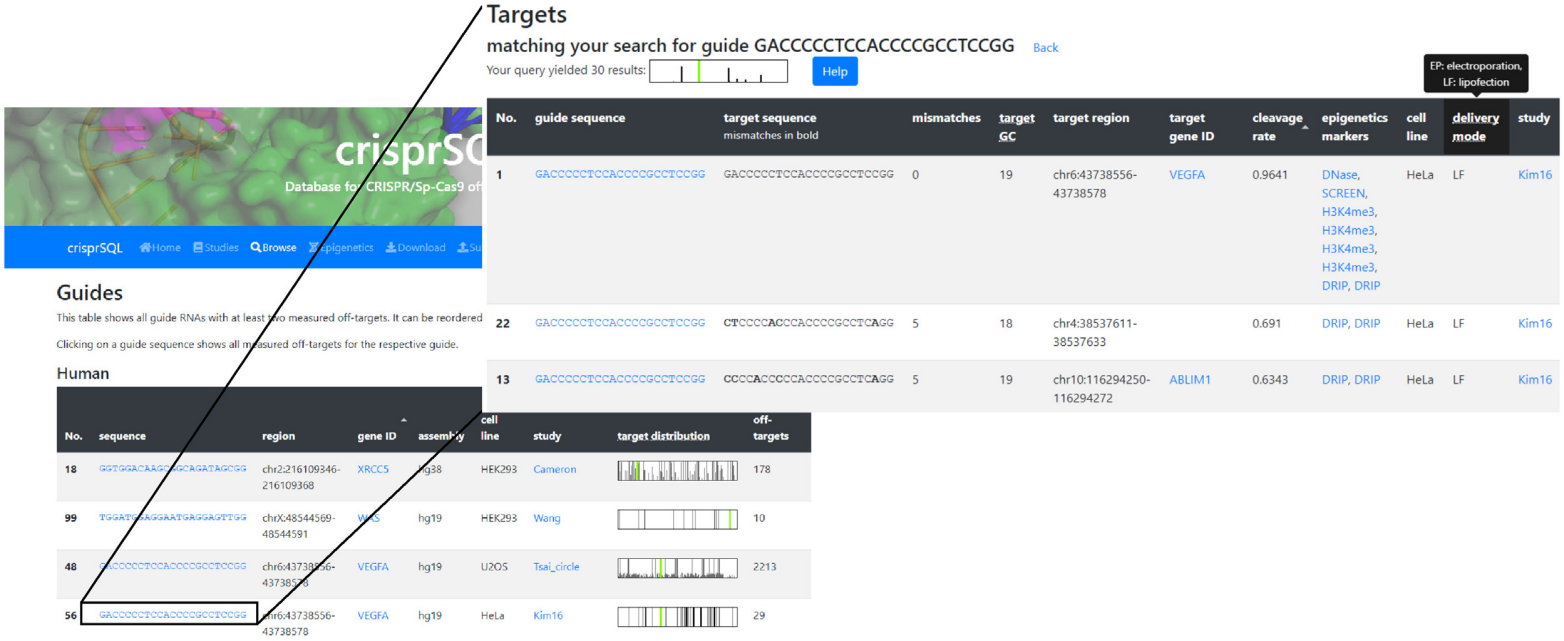
Web interface of the database, showing a list of all included gRNA sequences and which, if any, GENCODE gene name they target. Their respective target distributions across the genome are visualised as a barcode-type histogram, allowing a first assessment of their reported specificity. Upon clicking on or searching for a specific guide (inset), the website shows its full reported off-target profile, i.e. gene names of target loci, cleavage rates and epigenetic markers at the target site, hyperlinked to the respective source. Clicking on a gene name opens the vicinity of the respective cut site in a genome browser.

Through use of a state-of-the-art front-end framework, the website is fully functional on phone and tablet screens as well. An overview of the included off-target studies shows the respective detection assays, number of involved guides and targets as well as the gene names which have been predominantly cleaved (Figure 2). It is possible to browse through all included guide sequences grouped by sequence and cell line (Figure 3) as well as search for guide or target sequences, GENCODE gene names or loci. Search results are subsequently shown in a table linking to the original publications, to a genome browser showing the vicinity of the cut site as well as to studies which have demonstrated epigenetic markers at the respective DNA loci. Hyperlinks to all involved study publications and epigenetic data repositories ensure transparency and precise traceability of the included data. The search result is visualised as a barcode-type histogram plot depicting the locations and observed cleavage rates of found targets across the genome. A similar barcode is given for the editing profile of each guide, allowing a first insight into its specificity. The database therefore lends itself as a powerful in-depth literature research tool for planning off-target studies, such as comparing known off-target performances of specific guides or finding guides whose off-target effects include a specific gene or locus.

The website also allows submission of off-target study results as text files containing guide-target pairs, cell line, measured cleavage rates and publication reference which will then be processed and integrated into the database. In this way, we hope to contribute to data visibility and accessibility in the genome engineering field, as well as model comparability and reproducibility in the area of off-target prediction algorithms.

## RESULTS AND DISCUSSION

We have established a data processing pipeline and database tool which fill the niche of a thoroughly curated and annotated collection of base-pair resolved CRISPR/Cas9 off-target cleavage assay data. Besides providing a comprehensive search tool for gRNA design on both base pair and gene level, it can act as a data source for both the experimental validation of off-target detection pipelines and for computational off-target cleavage prediction algorithms. This will support experimental research into gRNA design for a variety of applications in the wider gene editing and transcriptomics fields, as well as enhance transparency and reproducibility of computational studies.

As noted above, experimental protocols for the different assays differ considerably in the concentration ratios of Cas9- and gRNA-encoding plasmids as well as the timespan between cell transfection and harvest. GUIDE-seq (6), Digenome-seq (13) and CIRCLE-seq (20) require a considerably more intricate computational pipeline than other methods to extract enriched genomic intervals after alignment to a reference genome and check for nuclease-induced edit sites. These represent specific assumptions and normalisation steps, creating the need to further investigate when absolute cleavage frequency values are compared between studies, and to at least normalise the cleavage rate distribution for each single study with a monotone, nonlinear function when relative rankings of cleavage frequencies are desired. An example of such a normalisation is shown in Supplementary Figure S1.

The at times incomplete validation of *in vitro* off-target effects in cells further complicates the comparison of cleavage sites between studies. For a direct comparison of sets of cleavage assays regarding their relative performance, we refer to references (17,20,23).

### Future developments

We invite submissions of appropriately resolved cleavage frequency data by experimental authors, which will be run through our annotation pipeline and included in the website in regular intervals. Another promising addition will be to include MNase-seq data (47) as an epigenetic marker gained on the respective cell lines in order to quantify nucleosome occupancy, which has been shown to correlate with cleavage frequency (48).

We further envision to extend the database by appropriately annotated studies targeting high-fidelity Cas9 nuclease variants (18) and nucleases from different organisms (14), as well as Cas9 off-target studies on different vertebrate organisms.

In order to provide a one-stop experience for off-target effects, we envision the inclusion of a state-of-the-art cleavage prediction algorithm which can provide predicted off-target effects next to measured off-target effects for a given gRNA.

## DATA AVAILABILITY

The crisprSQL database is available at http://www.crisprsql.com, where the full data set can be downloaded in csv format. Users are not required to log in to access any of the database features.

## Supplementary Material

gkaa885_Supplemental_File
