# Supplementary material for: crisprSQL: a novel database platform for CRISPR/Cas off-target cleavage assays
Source: Nucleic Acids Res. 2020 Oct 21;49(D1):D855–61. doi: 10.1093/nar/gkaa885 (PMC7778913; doi:10.1093/nar/gkaa885)
Supplement: gkaa885_Supplemental_File [file gkaa885_supplemental_file.pdf]

Cleavage Frequency Definition

Where mutation frequencies, indel frequencies or similar terms were given, we used these values for the cleavage frequency value. In case only relative read counts were given, cleavage frequency was calculated as the read ratio relative to the sum of reads for a given guide.

Data Normalisation

In order to compare cleavage frequencies across studies, references (1) and (2) suggest transforming cleavage rates to approximate a Gaussian with zero mean and variance σ^2=2 using the nonlinear Box–Cox transformation (3). Values above and below $\pm 2\sigma$ should be capped in order to achieve a fixed value range. As a logarithmic transformation, it is only valid for values larger than zero, which is why measured cleavage rates below the lowest reported measurement accuracy (10^{-5} in our case) should be manually transformed to -2σ (this should only apply to a select few data points). Supplementary Figure 1 exemplifies a transformation of this kind, which under certain circumstances allows the comparison of cleavage frequencies between studies and is essential e.g. for the assembly of a cross-study training set for off-target cleavage prediction algorithms.

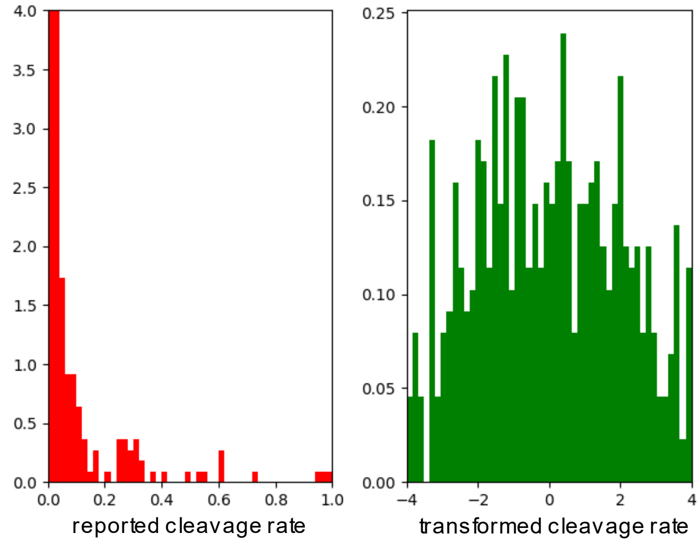

Supplementary Figure 1: Nonlinear transformation of the cleavage rate distribution at the example of study (4). Red bars (left) indicate the distribution before transformation, green bars after (right). The transformed distribution approaches a Gaussian with mean zero and variance σ^2=2 as desired, with values at $\pm$4 overemphasised due to capping.

References

(1) Listgarten, J.,Weinstein, M., Kleinstiver, B. P., Sousa, A. A., Joung, J. K., Crawford, J., Gao, K., Hoang, L., Elibol, M., Doench, J. G. et al. (2018) Prediction of off-target activities for the end-to-end design of CRISPR guide RNAs. *Nat. Biomed. Eng.*, **2**, 38–47.

(2) Wang, J., Xiang, X., Cheng, L., Zhang, X. and Luo, Y. (2019) CRISPR-GNL: an improved model for predicting CRISPR activity by machine learning and featurization. biorXiv doi: https://doi.org/10.1101/605790, 11 April 2019, pre-print: not peer-reviewed.

(3) Box, G. E. P. and Cox, D. R. (1964) An Analysis of Transformations. *J. R. Stat. Soc. B*, **26**, 211–252.

(4) Tsai, S. Q., Zheng, Z., Nguyen, N. T., Liebers, M., Topkar, V. V., Thapar, V., Wyvekens, N., Khayter, C., Iafrate, A. J., Le, L. P. et al. (2015) GUIDE-seq enables genome-wide profiling of off-target cleavage by CRISPR-Cas nucleases. *Nat. Biotechnol.*, **33**, 187–198.

Tables

Database field	GU_allowed	pos_weight	pam_corr	grna_folding	dna_opening	dna_pos_wgh
energy_1	False	False	False	True	False	False
energy_2	False	True	True	False	True	True
energy_3	False	True	False	False	True	True
energy_4	False	False	True	False	False	False
energy_5	False	False	False	False	False	True

Supplementary Table 1: Arguments for the crisprspec.get_eng() function with which CRISPRspec energy values have been retrieved.

No.	study	assay type	cell line
1	SCREEN ENCODE v4	DNase	embryo
2	SCREEN ENCODE v4	H3K4me3	embryo
3	SCREEN ENCODE v4	H3K4me3	HAP1
4	SCREEN ENCODE v4	CTCF	HEK293
5	SCREEN ENCODE v4	CTCF	HeLa
6	SCREEN ENCODE v4	DNase	HeLa
7	SCREEN ENCODE v4	H3K4me3	HeLa
8	SCREEN ENCODE v4	CTCF	K562
9	SCREEN ENCODE v4	DNase	K562
10	SCREEN ENCODE v4	H3K4me3	K562
11	GSM2871902	H3K4me3	HAP1
12	GSE68948	DRIP	HEK293
13	GSM683769	RRBS	HEK293
14	GSM720354	RRBS	HEK293
15	GSM720355	RRBS	HEK293
16	GSM2711412	H3K4me3	HEK293
17	GSM2902639	DNase	HEK293
18	ENCFF001TOL	RRBS	K562
19	ENCFF001TOM	RRBS	K562

No.	study	assay type	cell line
**20**	GSM683780	RRBS	K562
**21**	GSM683825	RRBS	K562
**22**	GSM683856	RRBS	K562
**23**	GSM1720619	DRIP	K562
**24**	GSE87831	DNase	U2OS
**25**	GSE87831	DNase	U2OS
**26**	GSM1296149	CTCF	U2OS
**27**	GSM1683507	DRIP	U2OS
**28**	GSM3147770	H3K4me3	U2OS
**29**	GSM3196008	DRIP	U2OS
**30**	ENCFF813SKE	DNase	HAP1
**31**	ENCFF944IGS	DNase	HAP1
**32**	ENCFF018VMV	CTCF	HEK293
**33**	ENCFF127KSH	DNase	HEK293
**34**	ENCFF781HLM	H3K4me3	HEK293
**35**	ENCFF781HLM	H3K4me3	HEK293
**36**	ENCFF148POZ	H3K4me3	K562
**37**	ENCFF251TOG	H3K4me3	K562
**38**	ENCFF500EUY	H3K4me3	K562
**39**	ENCFF616DLO	H3K4me3	K562
**40**	ENCFF718PBW	DNase	K562
**41**	ENCFF843VHC	CTCF	K562
**42**	ENCFF875WSG	DNase	K562
**43**	ENCFF961SPZ	H3K4me3	K562
**44**	ENCFF001YOC	DNase	embryo

No.	study	assay type	cell line
**45**	ENCFF940SWY	DNase	embryo
**46**	ENCFF041TGX	DNase	embryo
**47**	ENCFF357JNZ	H3K4me3	embryo
**48**	ENCFF940SWY	CTCF	embryo
**49**	ENCFF915PWP	CTCF	HeLa
**50**	ENCFF474KGT	CTCF	HeLa
**51**	ENCFF001TMU	RRBS	HeLa
**52**	ENCFF001TMV	RRBS	HeLa
**53**	ENCFF351NVF	DNase	HeLa
**54**	ENCFF982QSW	H3K4me3	HeLa
**55**	ENCFF542GBR	H3K4me3	HeLa
**56**	ENCFF201KBA	H3K4me3	HeLa
**57**	ENCFF102MXI	H3K4me3	HeLa
**58**	GSM2668157	DRIP	HeLa
**59**	GSM2452072	DRIP	HeLa
**60**	ENCFF331BAX	CTCF	HeLa
**61**	ENCFF862FGB	CTCF	HeLa
**62**	ENCFF095GJF	DNase	HeLa
**63**	ENCFF186BOU	DNase	HeLa
**64**	ENCFF879GTR	DNase	HeLa
**65**	ENCFF029YMX	H3K4me3	HeLa
**66**	ENCFF850XBJ	H3K4me3	HeLa
**67**	ENCFF336AJX	H3K4me3	HeLa
**68**	ENCFF479ZEP	H3K4me3	HeLa
**69**	ENCFF455VLU	DNase	HAP1

Supplementary Table 2: Epigenetic assay files currently included in the database, including the assay type and cell line to which they apply. This overview is reproduced from http://crisprsql.com/epigen.php.
